# Enabling Next-Generation Mass Spectrometry-Based Proteomics: Standards, Proteoform Resolution, and FAIR, Reproducible, and Quantitative Analysis

**DOI:** 10.3390/proteomes14020020

**Published:** 2026-04-21

**Authors:** Rui Vitorino

**Affiliations:** 1 iBiMED, Department of Medical Sciences, University of Aveiro, 3810-193 Aveiro, Portugal; rvitorino@ua.pt; 2 RISE, Department of Surgery and Physiology, Faculty of Medicine, University of Porto, 4099-002 Porto, Portugal

**Keywords:** FAIR infrastructures, proteoform-centric biology, AI/ML

## Abstract

Recent advances in mass spectrometry, data-independent acquisition, proteoform-resolving workflows, and multi-omics integration have significantly expanded the scale and scope of proteomics. However, the reuse and translational application of these datasets are limited by inconsistent standards, insufficient metadata, and inadequate computational interoperability. Proteoform-centric approaches provide higher molecular resolution by capturing intact protein variants and patterns of post-translational modification. Computational methods, including selected applications of machine learning and large language models (LLMs), are increasingly used for tasks such as spectral prediction and pattern discovery in clinical proteomics datasets. Despite these advancements, FAIR (Findable, Accessible, Interoperable, and Reusable) data practices, proteoform biology, and AI analytics are often pursued independently. This work presents an integrated framework for next-generation proteomics in which standardization and FAIR (Findable, Accessible, Interoperable, and Reusable) principles establish machine-actionable foundations for proteoform-resolved analysis and computational inference. It examines community efforts to promote data sharing and interoperability, as well as strategies for characterizing proteoforms using bottom-up, middle-down, and top-down approaches. It also highlights emerging AI and ML applications within the proteomics workflow. The framework emphasizes the importance of treating proteoforms as primary computational entities and adopting FAIR practices during data collection to enable reproducible and interpretable modeling. Finally, it introduces an architectural model that integrates FAIR infrastructures and proteoform resolution. In addition, practical recommendations for making AI-ready proteomics, including a minimal community checklist to support reproducibility, benchmarking, and translational scalability, are provided.

## 1. Introduction

Proteomics has reached an unprecedented level of scale and analytical depth. The measurable proteome has expanded significantly due to advances in high-resolution mass spectrometry, data-independent acquisition (DIA), native and top-down workflows, and multi-omics integration [1,2,3]. Modern research routinely generates terabyte-scale datasets from multi-center cohorts, longitudinal clinical trials, and systems-level biological studies. The ability to measure proteins with greater sensitivity, dynamic range, and throughput is no longer the primary challenge in this field [4,5,6].

Instead, the exponential growth of proteomic data has exposed structural bottlenecks that now define the frontier of progress. Reproducibility across laboratories, interoperability between analytical platforms, harmonization of metadata, and long-term dataset reusability remain inconsistent. Without standardized formats, controlled vocabularies, traceable spectral evidence, and robust quality control frameworks, proteomics data risk becoming computationally opaque and biologically underutilized. As a result, the principles of FAIR data stewardship (Findable, Accessible, Interoperable, and Reusable) have shifted from being aspirational goals to foundational requirements. In modern proteomics, FAIR is not just a guideline for open science; it is essential for large-scale analytics and meaningful secondary data reuse [7,8,9,10].

At the same time, the biological resolution of proteomics continues to improve. Conventional peptide-centric bottom-up workflows have enabled remarkable proteome coverage; however, they often merge biologically distinct molecular entities into aggregated protein groups. Proteoform-centric strategies, such as top-down, middle-down, and advanced PTM-aware approaches, address this limitation by identifying intact protein variants defined by sequence variation, post-translational modifications, truncations, and combinatorial modification states [2,11,12,13]. This shift is more than a technical advance; it represents a conceptual change in how it measures the true molecular complexity of the proteome, as biological function is often proteoform-specific.

Certain proteomics tasks, including spectral prediction, peptide property estimation, and pattern discovery in large clinical datasets, have benefited from computational approaches such as machine learning and newly developed large language model (LLM)-based frameworks. However, these applications remain primarily task-specific and depend heavily on standardized, high-quality data. Most existing implementations do not yet provide cross-domain inference or generalized molecular reasoning, highlighting the need for structured, FAIR-compliant datasets to support reliable computational modeling [14,15]. Despite rapid progress in data standardization, proteoform-centric biology, and AI-driven analytics, these areas are typically addressed in isolation. FAIR-focused reviews emphasize repositories and metadata frameworks, while the proteoform-centric literature centers on instrumentation and fragmentation strategies. AI in proteomics is primarily discussed in terms of algorithm performance and benchmarking. While these perspectives are valuable, they remain siloed [16,17,18]. None address how proteoform-resolved molecular entities achieve machine-actionability across studies, how FAIR infrastructures can directly support AI-driven causal inference, or how experimental workflows must adapt to enable proteoform-indexed clinical reporting. No single area can resolve these challenges. They require a unified architectural perspective that integrates data standards, molecular resolution, and computational inference.

This review addresses that gap. It argues that the advancement of next-generation proteomics depends on the coordinated evolution of three interconnected pillars: (1) robust standards and FAIR data practices that ensure datasets are machine-actionable, interoperable, and reproducible; (2) proteoform-centric analytical strategies that capture biologically meaningful molecular diversity beyond peptide aggregation; and (3) intelligent computational frameworks that can scale, integrate, and interpret complex, multi-layered proteomic information. These are not equivalent innovations, which is crucial. FAIR infrastructures enable reliable AI training and cross-domain study comparisons. Proteoform-aware representations restore biological accuracy and enhance model interpretability. AI techniques increase the utility of standardized, high-resolution datasets through predictive modeling and systems integration [19,20].

**Figure 1:**
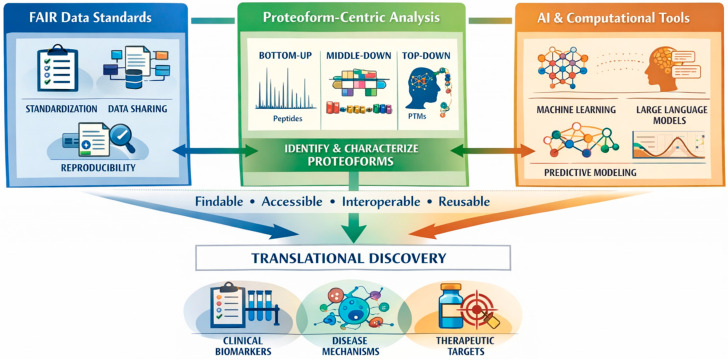
Architecture of next-generation proteomics: from FAIR data to proteoform biology and AI-driven discovery. Next-generation proteomics emerge from the co-development of FAIR infrastructure, proteoform-level molecular resolution, and AI-enabled analytics.

Based on this synthesis, a new structure for the field is proposed (Figure 1). Proteomics should become a field that is machine-ready and proteoform-aware. In this approach, proteoforms serve as the basic unit of computation, and FAIR principles are integrated at the point of data acquisition rather than being added later during deposition. Here, AI readiness is a design requirement, not an afterthought. This shift transforms proteomics from a field centered on measurements to one that uses computational methods to study molecular systems [21,22]. If this architectural change is correct, it should produce measurable results. By 2030, most major clinical proteomics trials in cancer, heart disease, and metabolic disease will report proteoform-indexed outputs instead of protein-group summaries. Additionally, AI models using proteoform-resolved inputs will outperform protein-group-based models in pathway inference, survival prediction, and cross-cohort generalization benchmarks. These predictions are empirically testable and provide specific criteria for evaluating the proposed framework. This framework suggests a possible direction for the field, but practical and conceptual problems remain to be solved. To create a machine-ready proteomics ecosystem, we need data standards that are compatible across platforms, robust benchmarking datasets, and reproducible computational pipelines. Additionally, integrating proteoform-level data into standard workflows requires balancing depth of analysis with scalability and robustness. This shift should not be viewed as an immediate transformation but as a gradual evolution that relies on coordinated progress in standardization, experimental design, and computational validation.

## 2. Standardization, Data Sharing, and FAIR Proteomics

The rapid expansion of proteomic data production has outpaced the development of analytical and reporting methodologies. Modern mass spectrometry platforms can now generate deep and quantitative proteomes with ease. However, the long-term scientific value of these datasets depends on their reproducibility, interoperability with other datasets, and reusability. Standardization has evolved from technical convenience to a fundamental requirement for scalable proteomics [23,24]. The FAIR principles provide a framework for addressing these challenges. In proteomics, FAIR extends far beyond simply depositing data in a database. It encompasses experimental metadata, traceability of spectral evidence, quality control metrics, and machine-readable result formats. Increasingly, FAIR is viewed as machine-actionable, reflecting the growing need for automated reanalysis, benchmarking, and integration with AI pipelines [25].

Community-driven initiatives have established an ecosystem of interoperable formats and controlled vocabularies for raw data, identifications, quantification, spectral libraries, quality control, and proteoform encoding. Simultaneously, federated repository infrastructures now store millions of datasets from diverse organisms, technologies, and experimental designs, enabling secondary analysis, method comparison, and large-scale meta-studies [16]. These advances facilitate global data sharing and computational reuse. Nevertheless, broad data availability does not guarantee reusability. A persistent issue with public proteomics datasets is incomplete metadata, experimental annotation, and quality reporting. Missing information about sample origin, acquisition, or processing often hinders reproducibility and study integration. In response, recent standardization efforts have increasingly focused on structured experimental descriptors, interoperable quality control reporting, and explicit links between biological conclusions and primary spectral evidence.

These challenges are particularly significant for DIA workflows and large-scale clinical proteomics, where minor differences in instrument performance or analytical pipelines can introduce systematic bias. Standardized reporting and harmonized quality metrics are essential not only for reproducibility but also to ensure that downstream computational models learn biological signals rather than technical artifacts [26,27,28]. At this intersection, AI and FAIR proteomics converge. For machine learning models to function across laboratories and platforms, they require large, diverse, and well-annotated datasets. Thus, FAIR compliance is essential for robust AI training, testing, and deployment. Proteomics datasets remain difficult for automated pipelines to process due to a lack of standardized metadata, traceable spectral evidence, and interoperable formats, limiting the scalability of data-driven discovery.

Standardization is also critical for proteoform-centric analysis. Representing intact protein variants, such as post-translational modifications, truncations, and sequence changes, requires machine-readable encoding schemes that are consistent across tools and repositories. Recent advances in proteoform representation now allow proteoforms to be treated as first-class computational objects rather than mere annotations, enabling integration with structural biology, pathway analysis, and AI-based modeling [29,30].

Overall, these developments signal a shift from generating isolated datasets to building interoperable, machine-actionable proteomics ecosystems. FAIR infrastructures are now central, defining the standards for large-scale, proteoform-resolved biology and advanced analytics. As proteomics becomes increasingly integrated with systems biology, clinical translation, and AI, standardization serves as the critical link between measurement, interpretation, and reuse. This change in architecture directly affects translation. Consider a group of people with heart failure from multiple centers studied using current proteomics methods. Peptide-level DIA outputs are now combined into protein groups, stored with various types of metadata, and analyzed using statistical models specific to each cohort. In machine-ready, proteoform-aware architecture, FAIR principles are applied at acquisition, intact proteoforms are clearly encoded, and datasets are designed to interoperate across sites. Proteoform-resolved inputs are then integrated into shared knowledge graphs and AI models trained at different centers [31,32]. This enables pathway-level inferences and allows patients to be grouped in ways that extend beyond individual cohorts. Proteomics has shifted from retrospective data analysis to a continuously learning system, with molecular resolution, computation, and clinical interpretation evolving together. Although significant progress has been made, current standards remain largely optimized for data deposition rather than inference. Formats for identifications and proteoform representation are rarely considered native substrates for AI pipelines or clinical reporting systems. What is still needed is a FAIR-by-design acquisition layer that integrates spectral evidence, experimental metadata, and proteoform objects at the source. This would make automated reanalysis, cross-cohort learning, and proteoform-indexed outcomes standard components of proteomics workflows. In the context of quantitative proteome analysis, targeted mass spectrometry approaches such as selected reaction monitoring (SRM) and parallel reaction monitoring (PRM) play a critical role in validation and clinical translation, offering high sensitivity and reproducibility for predefined targets. In addition, isobaric labeling strategies such as tandem mass tag (TMT)-based proteomics enable multiplexed quantitative analysis across multiple samples, supporting large-cohort and comparative studies. These quantitative methodologies are essential components of standardized proteomics workflows and complement discovery-driven approaches.

One of the most important steps to ensure that proteomics is reproducible and that data can be reused is to establish minimum information standards for reporting experiments. In other fields, such as quantitative PCR, the MIQE (Minimum Information for Publication of Quantitative Real-Time PCR Experiments) guidelines have provided a widely used framework to ensure that sufficient experimental detail and metadata are included to assess the quality, reproducibility, and interpretability of the data [33]. A similar approach is highly beneficial for mass spectrometry-based proteomics, especially for large-scale and quantitative studies. As noted above, dataset quality is a key factor in determining the reliability of downstream computational analyses, such as statistical modeling and machine learning. However, inconsistencies in sample preparation, acquisition parameters, data processing workflows, and annotation standards continue to hinder reproducibility and cross-study integration. It should be proposed that a “Minimum Information for Proteomics Experiments” (MIPE) framework be considered. This framework should cover key areas such as: (i) sample metadata (biological source, clinical annotation, pre-analytical variables), (ii) experimental design (replication, controls, randomization), (iii) mass spectrometry acquisition parameters (instrument type, fragmentation method, DIA/DDA settings), (iv) data processing workflows (search engines, FDR thresholds, normalization strategies), and (v) quantitative reporting standards (absolute vs. relative quantification, batch correction, missing value handling). Adopting these standardized reporting guidelines would significantly improve transparency, enable rigorous quality control, and facilitate dataset integration. This would support the development of robust and reproducible proteomics pipelines that adhere to FAIR principles.

## 3. Proteoform-Centric Workflows: From Peptide Aggregation to Intact Molecular Resolution

It is important to distinguish between classical top-down proteomics and mass spectrometry–intensive top-down proteomics (MSi-TDP) (Table 1). While top-down approaches can, in principle, analyze high-molecular-weight proteins, MSi-TDP workflows applied to complex proteomes are currently constrained by signal complexity, ionization efficiency, and fragmentation efficiency. Therefore, achievable molecular weight ranges must be interpreted in the context of sample complexity and analytical depth. Traditional bottom-up proteomics has achieved remarkable proteome coverage and quantitative depth by enzymatically cleaving proteins into peptides before mass spectrometric analysis. This peptide-focused approach underpins most large-scale studies and remains essential for high-throughput discovery. However, it inherently disrupts biological context. PTMs, sequence variants, truncations, and combinatorial modification states are distributed among different peptides and later computationally reassembled into protein groups. In this process, distinct molecular entities with potentially different functions are often combined into a single abundance estimate, making it difficult to discern specific functional roles [34,35,36]. The concept of the proteoform reframes this challenge. A proteoform is a specific molecular version of a protein arising from genetic variation, alternative splicing, and post-translational changes. Because biological activity is often specific to individual proteoforms, especially in signaling, chromatin regulation, and disease-associated pathways, measuring intact proteoforms represents a conceptual shift from approximate protein quantification to direct assessment of functional molecular states [2,37,38].

Proteoform-centric strategies include a variety of analytical applications, but they differ greatly in their ability to achieve complete proteoform resolution. Bottom-up workflows use enzymatic digestion and infer proteoforms indirectly through peptide-level PTM mapping and computational assembly. Middle-down methods also rely on proteolysis, typically employing different or fewer cleavage methods to generate larger fragments that preserve regional modification context. Although middle-down is often considered an intermediate approach, it is still based on digestion and therefore shares the conceptual and inferential limitations of bottom-up proteomics. Mass spectrometry–intensive top-down proteomics (MSi-TDP), by contrast, directly analyzes intact proteins, enabling clear identification of combinatorial modification states and sequence variants. However, current MSi-TDP implementations remain limited in proteome-wide depth and molecular weight range. Importantly, the effective molecular weight range of MSi-TDP is strongly dependent on sample complexity. While individual proteoforms exceeding 100 kDa can be analyzed under optimized or native conditions, routine proteome-scale applications in complex biological matrices are typically restricted to proteins below ~30–50 kDa. Although such studies demonstrate enhanced sequence coverage for selected proteins within the routine analytical range of current MSi-TDP, full proteome-scale intact coverage remains technically challenging. As a result, no single modality currently achieves both broad coverage and complete molecular fidelity. Deep proteoform analysis increasingly relies on integrative workflows that combine high-resolution intact proteoform characterization with subsequent digestion-based LC–MS/MS methodologies, enabling both molecular specificity and proteome-scale depth [39]. However, proteoform-centric proteomics also introduces important limitations. Compared with peptide-centric workflows, proteoform identification is often associated with reduced sensitivity, lower proteome coverage, and increased uncertainty in identification confidence. In complex biological samples, the combinatorial diversity of post-translational modifications further complicates proteoform assignment and quantification. As a result, peptide-centric approaches remain more robust and scalable for large-cohort studies, particularly in clinical settings. A key challenge is therefore balancing molecular specificity with analytical depth.

**Figure 2:**
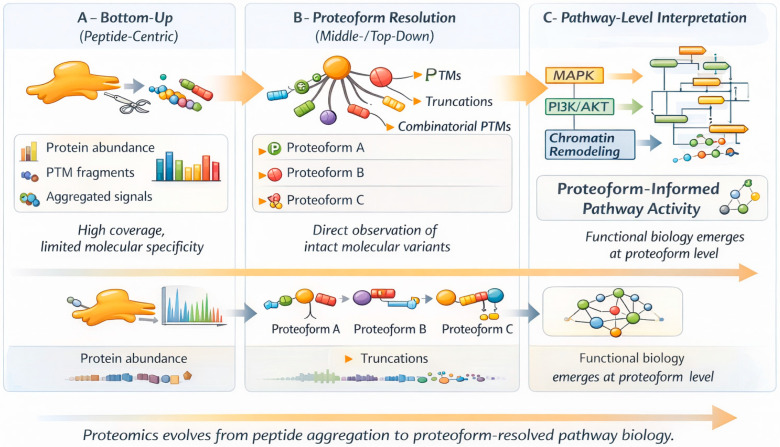
Increasing biological resolution across proteomic modalities.

Community efforts have accelerated progress across these areas. Standardizing methods, adopting native top-down workflows, and conducting cross-laboratory comparisons have all enhanced analytical rigor (Figure 2). Simultaneously, advances in machine-readable proteoform representation now allow complex molecular states to be consistently encoded across tools and repositories. These developments are essential for integrating proteoform data with structural biology, pathway analysis, and computational frameworks. However, the shift to proteoform-aware proteomics is not solely technical; it reflects a deeper computational need. If proteoforms are the true units of biological function, they must be stored as first-class data objects that are traceable, interoperable, and reusable within FAIR-compliant infrastructures. Currently, intact molecular variants are often resolved experimentally but then aggregated into protein groups for downstream analysis. This re-aggregation undermines the biological specificity that proteoform-centric workflows seek to restore. From a systems perspective, proteoform resolution provides AI with the detailed information needed for meaningful inference. Models trained on aggregated protein abundances will always produce blurred representations of cellular states. In contrast, proteoform-resolved datasets retain functional molecular distinctions, enabling more accurate pathway modeling, network reconstruction, and phenotype association. In this way, proteoform-centric workflows are a critical link between FAIR data infrastructures and advanced computational interpretation. Although methods continue to improve, proteoforms are still not widely used as primary computational entities in most analytical pipelines [40,41]. To bridge this gap, proteoform objects must be systematically encoded, indexed, and reused across repositories, knowledge graphs, and inference frameworks. Only then can proteomics fully transition from peptide aggregation to proteoform-resolved, computationally integrated molecular systems biology. In practice, achieving this transition will require hybrid analytical architectures that integrate intact proteoform interrogation with complementary digestion-based depth, rather than reliance on any single modality alone. Only then can proteomics fully transition from peptide aggregation to proteoform-resolved, computationally integrated molecular systems biology. Several new mass spectrometry-based technologies are transforming proteomics beyond the traditional bottom-up, middle-down, and top-down methods. Spatial proteomics enables the mapping of protein distributions within tissue architecture, providing context-specific molecular insights essential for understanding disease microenvironments. Single-cell proteomics is advancing toward elucidating cellular heterogeneity, although challenges remain in sensitivity, quantification robustness, and throughput. Cross-linking mass spectrometry (XL-MS) provides information about protein–protein interactions and the organization of proteins into complexes, linking proteomics to structural biology. Targeted mass spectrometry techniques, such as SRM/MRM and PRM, remain crucial for validation, clinical translation, and quantitative reproducibility. These complementary modalities strengthen the analytical framework of mass spectrometry-based proteomics and are essential for bridging discovery and translational applications.

In proteomics, computational methods are primarily used for specific analytical tasks, such as peptide identification, spectral prediction, retention time estimation, and interference correction in DIA workflows (Table 2). Recently, LLMs and similar architectures have shown promise in spectral prediction and pattern discovery within large clinical proteomics datasets, particularly when integrated with standardized repositories. However, these methods still depend on data quality and cannot yet draw general biological conclusions or mechanistic inferences. As a result, their utility is limited even with FAIR-compliant, well-annotated datasets. In this context, computational methods should be considered ancillary tools that support data interpretation rather than independent drivers of biological discovery.

## 4. Intersecting Themes and Future Directions: Toward Machine-Ready, Proteoform-Aware Proteomics

Proteomics is undergoing a structural transformation. What began as an experimental field focused on identifying proteins has evolved into a data-driven science operating at the scale of entire populations. However, advances in mass spectrometry alone are not sufficient to meet the demands of modern biology and translational research. The next phase in proteomics depends on the coordinated development of three interdependent layers: FAIR data infrastructures, proteoform-resolved molecular biology, and advanced computational analytics. These are not isolated or novel concepts occurring independently; they form a tightly integrated ecosystem. FAIR infrastructures provide the machine-actionable foundation necessary for automated reanalysis and cross-study integration. Proteoform-centric workflows deliver the biological resolution required to capture the functional diversity of molecules. AI/ML synthesize these inputs to generate mechanistic and predictive insights. Stagnation in any one area impedes progress in the others [22,42].

A central principle of this framework is that standardization is no longer a peripheral concern; it is now essential to discovery. Community organizations such as the HUPO Proteomics Standards Initiative and data-sharing platforms within the ProteomeXchange Consortium have collaborated to establish the foundations for interoperable proteomics [16,43,44] (Table 3). Nevertheless, adoption remains inconsistent, particularly regarding metadata completeness, quality control reporting, and proteoform representation. Without these elements, datasets remain difficult to reprocess, benchmark, or integrate into AI pipelines. Simultaneously, the increasing emphasis on proteoform-level biology requires new approaches to computation and data management. Proteoforms must be treated as primary data objects, traceable, interoperable, and reusable, rather than as supplementary annotations to peptide-based outputs. This shift is necessary to connect molecular variation with disease mechanisms, phenotypes, and pathway activity. AI models trained solely on protein abundance data from diverse sources will always produce imprecise representations of cellular states. Proteoform-resolved inputs preserve functional specificity and provide more robust substrates for inference.

From a computational perspective, the field is approaching a critical inflection point. AI tools now surpass traditional methods in peptide identification, spectral prediction, and DIA quantification, but their broader impact is limited by heterogeneous datasets and insufficient benchmarks. To ensure results are transferable across laboratories, instruments, and clinical settings, we need standardized metadata, universally applicable quality metrics, and transparent model reporting. Without these foundational elements, AI may remain a collection of tools optimized for specific contexts rather than a unified approach to data analysis [18,45] (Table 3).

### 4.1. Proteomics in Clinical Trials: From Protein Groups to Indexed Endpoints for Proteoforms

Consider a multi-center heart failure [46,47] or cancer trial [48,49] using plasma proteomics as an exploratory endpoint. Currently, DIA data are aggregated into protein groups and compared to outcomes such as survival or treatment response. While statistically useful, this approach conflates distinct molecular states into averaged signals. With a machine-ready, proteoform-aware architecture, the analytical focus shifts from protein abundance to specific proteoform states. For example, analyses could target phosphorylated receptor variants, truncated signaling intermediates, or combinatorial PTM patterns associated with drug sensitivity. In cancer research, this enables models to distinguish between active and inactive MAPK or PI3K/AKT signaling branches based on proteoform ratios rather than total protein levels [50,51]. Proteoform-resolved inflammatory or extracellular matrix signatures could differentiate maladaptive remodeling from compensatory responses in heart failure cohorts, supporting patient stratification beyond the capabilities of standard biomarkers [52,53].

These proteoform-indexed features can serve as robust endpoints for AI models predicting treatment efficacy or patient survival. Proteoform-resolved models are expected to generalize better across cohorts than protein-group-based models because they capture pathway-relevant molecular states rather than cohort-specific abundance patterns. Crucially, these workflows also enable regulatory traceability. Model predictions can be linked directly to specific spectra, encoded proteoforms, and acquisition metadata, creating an auditable chain from raw data to clinical inference. This traceability is essential for deploying AI in regulatory settings.

### 4.2. Longitudinal Cohorts and Biobanks: Planning for AI in the Future

Longitudinal cohorts and population biobanks represent another type of translational scenario. In this context, proteomics data collected today will increasingly serve as inputs for AI models developed years from now. Currently, most work on biobank proteomics emphasizes data deposition rather than computational readiness [54,55]. Metadata are inconsistent, quality control metrics are not always recorded accurately, and molecular representations are designed for human readability rather than machine reusability. As a result, it will be very difficult for future AI models to interoperate and learn from each other across different groups.

FAIR-by-design acquisition, in contrast, standardizes experimental design, spectral evidence, quality metrics, and proteoform encodings at the source. This approach produces datasets that can be reused for new analytical paradigms. For example, plasma profiles resolved by proteoforms today could later be integrated with transcriptomic, imaging, or electronic health record data to train foundation models for risk prediction in multiple ways. Such analyses become unmanageable without machine-readable metadata and proteoform-aware representations. Within this framework, proteomics shifts from a static measurement layer to a continuously learning infrastructure that enables retrospective reanalysis, cross-cohort modeling, and mechanistic discovery at the population scale.

### 4.3. Effects on Deployment, Ethics, and Regulation

The convergence of FAIR infrastructures, proteoform resolution, and AI directly impacts regulatory and ethical considerations (Table 4). Clinical AI systems must be open, reproducible, and supported by data traceable to its source. Proteoform-aware workflows that maintain links between model outputs and primary spectral evidence establish a robust chain of evidence for audit and validation [56,57]. Standardized, interoperable data objects and formats facilitate independent model replication and reduce risks associated with opaque or irreproducible models. In this context, FAIR-by-design proteomics is not only a goal for open science but also the infrastructure that enables accountable clinical AI and also supports regulatory review, ethical deployment, and clinician trust.

### 4.4. Towards a Machine-Ready Proteomics Architecture

Several priorities now emerge:

1. FAIR-by-design experimental workflows that incorporate standardized metadata capture and quality control at acquisition, rather than retrofitting them at deposition.
2. Proteoform-aware data representations, enabling intact molecular entities to be queried, compared, and modeled across studies.
3. AI development grounded in benchmarks, using shared reference datasets and standardized evaluation metrics to ensure cross-platform performance and reproducibility.
4. Interpretable models that link proteoform changes to pathway alterations and phenotypic outcomes.
5. Feedback loops between AI and experiments, where model results inform targeted proteoform validation and experimental prioritization.

Even with these priorities, several issues must be addressed before proteomics can be considered truly machine-ready. These include the lack of universally accepted data standards across acquisition platforms, variability in data processing pipelines, and the limited availability of high-quality benchmarking datasets. Reproducibility between laboratories also remains poor, especially in large-scale clinical proteomics. If these problems are not resolved, computational models, including those using AI, may learn technical artifacts instead of biological signals. To make proteomics machine-ready, collaboration on standardization, validation, and cross-cohort reproducibility is essential.

These directions establish a new paradigm: proteomics as a machine-ready, biologically accurate, and computationally integrated field. In this framework, FAIR infrastructures enable proteoform biology, proteoform resolution enhances AI interpretability, and AI-driven inference accelerates discovery and translation. The field now requires architectural thinking, moving beyond incremental improvements to individual technologies. This means designing proteomics pipelines as end-to-end systems that support reproducibility, mechanistic insight, and scalable analytics. For next-generation proteomics to achieve its full scientific and clinical potential, this integration will be essential. Computational approaches in proteomics are primarily applied to well-defined analytical tasks, including peptide identification, spectral prediction, and quantitative signal extraction, particularly in data-independent acquisition workflows. More recent developments, including LLM-related approaches, have shown potential in spectral prediction and pattern discovery in large-scale proteomics datasets. However, these methods remain dependent on high-quality, standardized data and are not yet capable of generalized biological inference. Their impact is therefore closely linked to the availability of FAIR-compliant datasets and robust experimental design.

## 5. Conclusions

Proteomics is undergoing a structural transformation. Although advances in mass spectrometry, proteoform-resolving workflows, and artificial intelligence have enabled the analysis of larger datasets, their impact on real-world applications remains limited by inconsistent standards, low molecular resolution in downstream analysis, and poor compatibility across computing platforms. Overcoming these challenges requires more than incremental technical improvements; it calls for a fundamental rethinking of the field’s architecture. This study proposes that next-generation proteomics should be developed as a machine-ready, proteoform-aware discipline, where FAIR-by-design infrastructures provide machine-actionable data, proteoforms serve as the core biological and computational unit, and AI frameworks integrate molecular resolution with pathway- and phenotype-level inference. These components are interdependent: FAIR principles enable large-scale computation, proteoform resolution restores biological specificity, and AI transforms standardized, high-fidelity datasets into predictive and mechanistic insights.

Standardization thus becomes enabling infrastructure rather than supplementary support. For reproducibility, cross-study integration, and AI that meets regulatory requirements, it is essential to include structured metadata, quality control, and proteoform encoding at the point of data acquisition. Equally important is treating proteoforms as first-class computational entities to connect molecular variation with pathways and clinical outcomes. Without these foundations, AI is restricted to local optimization and cannot function as an integrative engine for molecular systems biology.

The implications for translation are substantial. Proteoform-indexed endpoints provide mechanistically grounded alternatives to protein-group summaries in clinical trials, improving patient stratification and model interpretability. FAIR-by-design proteomics transforms long-term cohorts and biobanks into future-proof resources for cross-cohort learning and multimodal AI. Simultaneously, spectral traceability and standardized data objects enable ethical clinical deployment that is open and verifiable.

Realizing this vision will require collective effort from the community. This includes implementing FAIR-by-design workflows, proteoform-aware data representations, validated and interpretable AI models, and feedback loops between computation and experimentation. If successful, proteomics can advance from descriptive measurement to a reproducible, computationally interoperable, and mechanistically precise foundation for systems biology and precision medicine. The choices made now regarding standards, molecular resolution, and advanced analytics will determine whether the field achieves this potential in the next decade.

## Figures and Tables

**Table 1:** Comparison of proteomics modalities (resolution-oriented framing).

Feature	Bottom-Up Proteomics	Middle-Down Proteomics	Top-Down Proteomics
Analyte	Peptides	Large proteolytic fragments	Intact proteins
Typical MW analyzed	0.5–3 kDa	~3–20 kDa (fragment range)	Typically <30–50 kDa in complex mixtures; higher MW achievable in targeted or simplified systems
Proteoform resolution	Indirect/inferred (assembly-based)	Fragment-resolved (digestion-dependent)	Direct (intact combinatorial states within working range)
PTM localization	Peptide-level	Regional (multi-site context within fragment)	Whole-protein (within accessible MW window)
Throughput	Very high	Moderate	Lower
Proteome coverage	Excellent (depth)	Moderate	Limited (proteome-scale depth constrained)
Quantification maturity	Very high (DIA/DDA established)	Developing	Emerging
Biological context preservation	Fragmented; computational reassembly required	Partial; retains regional modification context	Preserved molecular identity (within MW limits)
Instrumental complexity	Moderate	High	Very high
Computational burden	High	High	Very high
Best suited for	Large cohorts, discovery-scale quantification	Regional PTM mapping, targeted proteoform interrogation	Intact proteoform characterization, structural integration

Middle-down workflows remain fundamentally proteolysis-based and therefore share inferential assembly constraints with bottom-up proteomics, despite preserving larger sequence context. Comprehensive proteoform coverage at the proteome scale currently requires integrative strategies combining intact protein analysis with complementary digestion-based depth. Current mass spectrometry–intensive top-down proteomics (MSi-TDP) enables robust proteoform identification primarily in the ~5–30 kDa range for complex proteomes. Extended mass ranges (~50–70 kDa or higher) can be achieved under targeted, low-complexity, or native conditions.

**Table 2:** Current community efforts mapped to the three architectural pillars.

Initiative/Tool Class	FAIR Infrastructure	Proteoform Resolution	AI/ML Integration
HUPO-PSI Formats	✔	◐	✖
ProteomeXchange Resources	✔	◐	✖
Top-Down Proteomics Initiatives	◐	✔	◐
DIA Pipelines	◐	✖	✔
AI Identification Tools	✖	✖	✔
Clinical Proteomics Consortia	◐	✖	◐
Proposed Framework	✔	✔	✔

Legend: ✔ mature; ◐ emerging; ✖ largely absent.

**Table 3:** Minimal AI-Ready Proteomics Checklist (Stakeholder-Oriented).

Stakeholder	Actionable Requirement (Testable)
Data Producers (Labs/Core Facilities)	Deposit raw spectra + acquisition metadata at submission time
	Record experimental design in machine-readable form
	Provide QC metrics per run
	Encode PTMs/proteoforms using standardized identifiers
Repositories	Require structured metadata validation
	Support proteoform objects as primary entities
	Expose APIs for automated reuse
Tool Developers	Accept standardized formats (mzML/mzTab/ProForma)
	Output traceable spectral evidence
AI Modelers	Publish training datasets + schemas
	Separate training/validation/test cohorts
	Report model uncertainty
Journals and Funders	Mandate FAIR-at-acquisition
	Require proteoform-aware reporting where applicable

Datasets satisfying these criteria are FAIR, machine-actionable, and suitable for proteoform-aware AI modeling.

**Table 4:** Proteomics maturity model: from FAIR-Lite to machine-ready.

Level	Description	Concrete Criteria
Level 1—FAIR-Lite, Peptide-Centric	Traditional workflows	Peptide-level outputs only; metadata incomplete.
Level 2—Standardized	FAIR deposition	Structured datasets with basic metadata annotation and standard QC procedures (e.g., identification confidence, FDR control), but limited validation and reproducibility assessment.
Level 3—Proteoform-Aware	Molecular resolution	Proteoforms encoded; PTMs localized.
Level 4—Machine-Ready	Fully integrated	Fully standardized, interoperable, and high-quality datasets supported by rigorous quality control procedures, including advanced QC metrics, batch effect assessment, reproducibility testing, and cross-cohort validation. At this level, explicit validation frameworks are implemented to ensure robustness of computational and AI-driven analyses, including error detection mechanisms, benchmarking against reference datasets, and validation through experimental testing and/or independent published datasets. Feedback loops between computational models and experimental validation are integrated to continuously refine data quality and model performance.

Most current laboratories operate at Levels 1–2. Translational proteomics requires progression toward Level 4.

## Data Availability

No new data were created or analyzed in this study.
